# DEMENTIA STAGING, NEUROPSYCHIATRIC SYMPTOMS, AND DIVORCE OR SEPARATION IN LATE LIFE: A CASE CONTROL STUDY

**DOI:** 10.1093/geroni/igad104.0307

**Published:** 2023-12-21

**Authors:** Joan Monin, Gail McAvay, Emma Zang, Brent Vander Wyck, Carmen Carrión, Heather Allore

**Affiliations:** Yale School of Public Health, New Haven, Connecticut, United States; Yale University, New Haven, Connecticut, United States; Yale Sociology Department, Yale University, New Haven, Connecticut, United States; Yale School of Medicine, New Haven, Connecticut, United States; Yale School of Medicine, New Haven, Connecticut, United States; Yale University, New Haven, Connecticut, United States

## Abstract

Dementia can be difficult on marriages for many reasons, including but not limited to caregiving burden, loss of intimacy, and financial burden. This study examined the impact of dementia staging and neuropsychiatric behavioral symptoms on the likelihood of divorce or separation for older adult married couples. For this case-control study, we used data from the National Alzheimer’s Coordinating Center (NACC) Uniform dataset (UDS) versions 2 and 3. This dataset was from 2007 to 2021 and contains standardized clinical information submitted by NIA/NIH Alzheimer’s Disease Research Centers (ADRCs) across the United States (US). This data was from 37 ADRCs. We selected participants who were married or living as married/domestic partners at their initial visit. Cases were defined by a first divorce/separation occurring during the follow-up period, resulting in 291 participants. We selected 5 controls for each married/living as married case and matched on age. Conditional logistic regression estimated the association between overall Neuro Psychiatric Inventory (NPI) score and severity of NPI symptoms with case/control status, adjusted for education, the CDR® Dementia Staging Instrument score, living situation, symptom informant, sex, and race. Separate analyses were conducted for each symptom. Multiple comparisons were accounted for with the Hochberg method. Later stage of dementia was negatively associated with divorce/separation with an adjusted odds ratio. A higher overall NPI score was positively associated with divorce/separation. More severe ratings of agitation/aggression, depression/dysphoria, and disinhibition with divorce/separation were associated with greater odds of divorce/separation. Treating NPI symptoms may be away to protect relationships.

